# Dietary composition modulates brain mass and solubilizable Aβ levels in a mouse model of aggressive Alzheimer's amyloid pathology

**DOI:** 10.1186/1750-1326-4-40

**Published:** 2009-10-21

**Authors:** Steve Pedrini, Carlos Thomas, Hannah Brautigam, James Schmeidler, Lap Ho, Paul Fraser, David Westaway, Peter St George Hyslop, Ralph N Martins, Joseph D Buxbaum, Giulio M Pasinetti, Dara L Dickstein, Patrick R Hof, Michelle E Ehrlich, Sam Gandy

**Affiliations:** 1 Farber Institute for the Neurosciences, Jefferson Medical College, Philadelphia PA USA; 2 Departments of Neurology, Psychiatry, and Alzheimer's Disease Research Center, Mount Sinai School of Medicine, New York NY USA; 3 James J. Peters Veterans Affairs Medical Center, Bronx NY USA; 4 Department of Psychiatry, and Alzheimer's Disease Research Center, Mount Sinai School of Medicine, New York NY USA; 5 Centre for Research in Neurodegenerative Diseases, University of Toronto, Toronto, Canada; 6 Centre for Prions and Protein Folding Diseases, University of Alberta, Edmonton, Alberta, Canada; 7 University of Cambridge, Cambridge, UK; 8 Edith Cowan University, Joondalup, Western Australia, Australia; 9 Department of Neuroscience, and Alzheimer's Disease Research Center, Mount Sinai School of Medicine, New York NY USA; 10 Departments of Pediatrics and Neurology, and Alzheimer's Disease Research Center, Mount Sinai School of Medicine, New York NY USA

## Abstract

**Objective:**

Alzheimer's disease (AD) is a progressive neurodegenerative disease of the central nervous system (CNS). Recently, an increased interest in the role diet plays in the pathology of AD has resulted in a focus on the detrimental effects of diets high in cholesterol and fat and the beneficial effects of caloric restriction. The current study examines how dietary composition modulates cerebral amyloidosis and neuronal integrity in the TgCRND8 mouse model of AD.

**Methods:**

From 4 wks until 18 wks of age, male and female TgCRND8 mice were maintained on one of four diets: (1) reference (regular) commercial chow; (2) high fat/low carbohydrate custom chow (60 kcal% fat/30 kcal% protein/10 kcal% carbohydrate); (3) high protein/low carbohydrate custom chow (60 kcal% protein/30 kcal% fat/10 kcal% carbohydrate); or (4) high carbohydrate/low fat custom chow (60 kcal% carbohydrate/30 kcal% protein/10 kcal% fat). At age 18 wks, mice were sacrificed, and brains studied for (a) wet weight; (b) solubilizable Aβ content by ELISA; (c) amyloid plaque burden; (d) stereologic analysis of selected hippocampal subregions.

**Results:**

Animals receiving a high fat diet showed increased brain levels of solubilizable Aβ, although we detected no effect on plaque burden. Unexpectedly, brains of mice fed a high protein/low carbohydrate diet were 5% lower in weight than brains from all other mice. In an effort to identify regions that might link loss of brain mass to cognitive function, we studied neuronal density and volume in hippocampal subregions. Neuronal density and volume in the hippocampal CA3 region of TgCRND8 mice tended to be lower in TgCRND8 mice receiving the high protein/low carbohydrate diet than in those receiving the regular chow. Neuronal density and volume were preserved in CA1 and in the dentate gyrus.

**Interpretation:**

Dissociation of Aβ changes from brain mass changes raises the possibility that diet plays a role not only in modulating amyloidosis but also in modulating neuronal vulnerability. However, in the absence of a study of the effects of a high protein/low carbohydrate diet on nontransgenic mice, one cannot be certain how much, if any, of the loss of brain mass exhibited by high protein/low carbohydrate diet-fed TgCRND8 mice was due to an interaction between cerebral amyloidosis and diet. Given the recent evidence that certain factors favor the maintenance of cognitive function in the face of substantial structural neuropathology, we propose that there might also exist factors that sensitize brain neurons to some forms of neurotoxicity, including, perhaps, amyloid neurotoxicity. Identification of these factors could help reconcile the poor clinicopathological correlation between cognitive status and structural neuropathology, including amyloid pathology.

## Introduction

Alzheimer's disease (AD) is a neurodegenerative disease of the central nervous system characterized by an accumulation of extracellular and cerebrovascular amyloid and intracellular aggregated tau protein [1]. Amyloid deposits are composed of amyloid-β peptide (Aβ), a 4 kD fragment generated by proteolytic processing of the transmembrane amyloid precursor protein (APP) [2]. The "amyloid cascade hypothesis" is one popular model of AD pathogenesis, and amyloidogenic processing by γ-secretases is enhanced by mutations in either APP, presenilin 1, or presenilin 2. Among the two major species of Aβ, the C-terminally extended Aβ42 is more highly amyloidogenic than is the shorter but more abundant Aβ40. The ratio of Aβ42/Aβ40 determines the ratio of Aβ clearance vs accumulation, and Aβ aggregates or oligomers are believed to lead eventually to neuronal and synaptic dysfunction and neuronal death [2]. In addition to the deleterious amyloidogenic pathway, an additional non-amyloidogenic pathway has also been described. In this pathway, APP is first cleaved by one of several α-secretases (e.g., ADAM10, ADAM17) between positions 16 and 17 of the potential Aβ fragment, thereby precluding amyloidogenesis [2].

Extensive data support the notion that diets low in calories, cholesterol, and saturated fatty acids, but rich in vegetables, fruits, and fish, may delay the onset and/or slow the progression of clinical AD. The regulation of various secretases by dietary components is also well-documented (e.g., cholesterol regulation of γ-secretase; [3]). However, there have been no head-to-head studies employing diets high with various specified sources of calories to determine whether dietary composition has importance beyond the known effect of high caloric intake to exacerbate amyloid pathology [4]. Therefore, in the current study we evaluated the effects of 4 different diets in a murine model of AD (TgCRND8) that harbors 3 mutations in the APP gene (K670M/N671L and V717F)[5]. Using this mouse model of AD, we set out to determine whether different dietary compositions could modulate Aβ levels, brain mass, neuronal density, and/or tissue volume in 3 subregions of the hippocampus.

## Methods

### Mice and diets

TgCRND8 mice (n = 57) were kept in static cages under a 12 hour light/dark cycle. Cages were changed at least once each week, and the health of the mice was monitored daily. Mice were weaned at the age of 4 weeks and placed on one of four different diets: (1) regular or reference commercial chow, RC; (2) high fat/low carbohydrate custom chow (60 kcal% fat/30 kcal% protein/10 kcal% carbohydrate), HF/LC; (3) high protein/low carbohydrate custom chow (60 kcal% protein/30 kcal% fat/10 kcal% carbohydrate), HP/LC; or (4) high carbohydrate/low fat custom chow (60 kcal% carbohydrate/30 kcal% protein/10 kcal% fat), HC/LF (Table 1) until the age of 18 weeks. These custom diets were formulated by Research Diets, Inc. (New Brunswick, NJ, USA) in collaboration with Christopher O'Brien, Product Manager, Research Diets, Inc, so as to avoid diets associated with predictable organ toxicity. Mice were supplied with food and water *ad libitum *and were weighed weekly. At the age of 18 weeks, mice were sacrificed via isoflurane inhalation, and the brains were removed and weighed. The two cerebral hemispheres were separated for further experiments. One hemisphere was snap-frozen in isopentane while the other was immersion-fixed in 4% paraformaldehyde.

**Table 1:** Chow nutritional information and ingredients

		**High fat**		**High carbohydrate**		**High protein**		**Regular Chow**
		**g %**	**kcal %**	**g %**	**kcal %**	**g %**	**kcal %**	**kcal %**

Protein		39	30	29	30	65	60	29
Carbohydrate		13	10	58	60	11	10	58
Fat		35	60	4	10	14	30	13
Total			100		100		100	100
Total kcal/g		5.2		3.8		4.3		

**Ingredients**		g	kcal	g	kcal	g	m cal	
	
Casein, 80 mesh		300	1200	300	1200	600	2400	
L-Cystine		4.5	18	4.5	18	9	36	
Corn starch		0	0	264	1056	0	0	
Maltodextrin 10		80	320	35	140	80	320	
Sucrose		12.25	49	299.	1198	11.5	46	
Cellulose BW200		50	0	50	0	50	0	
Soybean Oil		25	225	25	225	25	225	
Lard		245	2205	20	180	110	990	
Mineral	Mix	10	0	10	0	10	0	
Dialcium		13	0	13	0	13	0	
Calcium		5.5	0	5.5	0	5.5	0	
Potassium		16.5	0	16.5	0	16.5	0	
Vitamin	Mix	10	40	10	40	10	40	
Choline		2	0	2	0	2	0	
Cholesterol		0	0	0.21	0	0.068	0	
	
Total		773.8	4057	1055	4057	942.62	4057	

For the Regular Chow (reference Rodent Chow 5010) the ingredients were: Protein 24.6%, Fat (ether extract) 4.8%, Fat (acid hydrolysis) 5.5%, Fiber (crude) 4.1%, Nitrogen-Free extract (by difference) 76.6%, Gross Energy kcal/g 4.14, Minerals and vitamins.

### Genotyping

Mice were subjected to a tail biopsy, and DNA was extracted using a Sigma DNA extraction kit (Sigma, St. Louis, MO) according to the manufacturer's instructions. The DNA was then amplified using APP-Hu-Forward primer 5'-CCG ATG ATG ACG AGG ACG AT-3' and APP-Hu-Reverse primer 5'-TGA ACA CGT GAC GAC GCC GA-3' in order to identify the 525 bp amplicon in TgCRND8 mice.

### Aβ ELISA

One cerebral hemisphere was homogenized 10% weight/volume (w/v) in tissue homogenization buffer (250 mM sucrose, 20 nM Tris-HCl pH 7.4, 1 mM EDTA, 1 mM EGTA + protease inhibitors), then a 200 μl aliquot of extract was added to a 440 μl aliquot of formic acid (FA) (minimum 95%), sonicated in ice for 1 minute and spun at 100,000 g for 1 hr at 4°C. Subsequently, a 210 μl aliquot was neutralized in 4 ml of FA neutralization buffer (1 M Tris base, 0.5 M Na_2_HPO_4_, 0.05% NaN_3_), stored at -80°C, and used for Aβ40 and Aβ42 evaluation by ELISA (The Genetics Company, Schlieren, Switzerland) according to the manufacturer's instructions. All values were then standardized to the protein concentration of the brain homogenate. Samples were analyzed in triplicate.

### Immunohistochemistry and plaque density quantification

The second, unfrozen hemisphere was fixed in 4% paraformaldehyde in PBS. Immunohistochemistry was performed on every 10^th ^of a series of 40 μm-thick sagittal sections and every 5^th ^of a series of 50 μm-thick coronal section. Sections were chosen from a random starting point and immunostained using anti-human APP antibody 6E10 (1:1,000) followed by a biotinylated monoclonal anti-mouse secondary antibody (1:200) protocol. For plaque density determination, each brain tissue section was traced at 2.5× using StereoInvestigator (MBF Bioscience, Williston, VT). Systematic-random samples from each brain tissue section were taken at a 20× magnification. Images were then acquired and volume and plaque density were measured using Image J v1.38e (). The results were expressed as mean ± standard error of the mean (SEM).

### Stereological analysis

Sections adjacent to those used for immunohistochemistry were used for Nissl staining. For stereologic analyses using the Optical Fractionator method, each section was viewed on an Olympus BX51 microscope at low magnification (4×/0.32 N.A. Plan-Apochromat) and the regions were contoured onto a live computer image using StereoInvestigator software. Counting of individual neurons was performed at a higher magnification (60×/1.4 N.A. Plan-Apochromat), using disector frames that were placed within each contour using a systematic-random design established by the software. For each section, the disector height was kept constant in the region of interest in each case.

### Reference volume estimation

The Cavalieri principle was utilized for regional volume estimation using a small 75 × 75 μm grid to obtain an unbiased stereologic estimate of the independent volumes of subregions of the hippocampus: CA1, CA3, and DG. CA2 was included within the CA1 contour for all analyses.

### Coefficients of error (CE)

The mean CEs for animals on the RC diet were 0.10, 0.12, and 0.10 for CA1, DG, and CA3, respectively. The mean CEs for animals on HF/LC diet were 0.12, 0.13, and 0.10, respectively. Mean CEs for animals on the HC/LF diet were 0.14, 0.14, and 0.11, respectively. The mean CEs for animals on HP/LC diet were 0.14, 0.15, and 0.14, respectively.

### Statistical analysis

ELISA of solubilizable Aβ40 and Aβ42, plaque density, weight, brain weight, and regional neuronal counts and volumes were analyzed using two-way analysis of variance (ANOVA) with diet and sex as the independent variables. Dunnett's multiple comparison procedure for pairwise comparison of three diets with RC was based on Dunnett's T3 procedure for unequal variances. For weight, a similar repeated measures ANOVA was performed to assess changes with age. Linear trends in weight were also analyzed. The subregions of the hippocampus were not included as independent variables due to the fact that the regions already have an inherent difference in cell density and volume, rendering any comparison between them within a single subject uninformative. α level was set at 0.05 for all analyses in the study, including the Dunnett multiple comparisons of RC with the other three diets.

### Attrition

Interindividual variations on Aβ levels in TgCRND8 mice of this age range can vary from 6- to 10-fold [5]. "Outlier" mice (i.e., with solubilizable Aβ levels two S.D. outside the mean of the other individuals of their respective groups; n = 6) were excluded from the analyses of solubilizable Aβ levels. Among the 51 mice remaining in the study, 9 were excluded from the brain/total weight analysis because a substitute operator's data were highly erratic and deemed unreliable. Consistent with the Chishti *et al*. data on premature death of TgCRND8 mice [5], 6-9 mice (29-39%) in each group died during the 18 wk trial (6 of 19 died on RC; 8 of 21 died on HF/LC; 7 of 24 died on HP/LC; 9 of 23 died on HC/LF).

## Results

The goal of this study was to evaluate the effect on Alzheimer's type neuropathology associated with one of four different diets: regular or reference commercial chow (58 kcal% carbohydrate/29 kcal% protein/13 kcal% fat), RC; high carbohydrate custom chow (60 kcal% carbohydrate/30 kcal% protein/10 kcal% fat), HC/LF; high fat custom chow (60 kcal% fat/30 kcal% protein/10 kcal% carbohydrate), HF/LC; high protein custom chow (60 kcal% protein/30 kcal% fat/10 kcal% carbohydrate), HP/LC; on solubilizable Aβ levels and neuronal loss in TgCRND8 plaque-forming mice. According to the two-way ANOVA, diet (as a general variable) played a role in determining both solubilizable Aβ40 levels (F_(3,43) _= 7.298, p < 0.0005) and solubilizable Aβ42 levels (F_(3,43) _= 13.978, p < 0.0005). Sex also played a role in solubilizable Aβ40 and Aβ42 levels with levels being higher in females than males (Aβ40, F_(1,43) _= 7.685, p = 0.008; Aβ42, F_(1,43) _= 11.835, p = 0.001). Interestingly, there was no interaction between diet and sex for either solubilizable Aβ40 (F_(3,43) _= 0.770, p = 0.517) or solubilizable Aβ42 (F_(3,43) _= 0.920, p = 0.439) demonstrating that the sex effect was similar within each diet, and, correspondingly, that the diet effect was similar within each sex.

**Figure 1:**
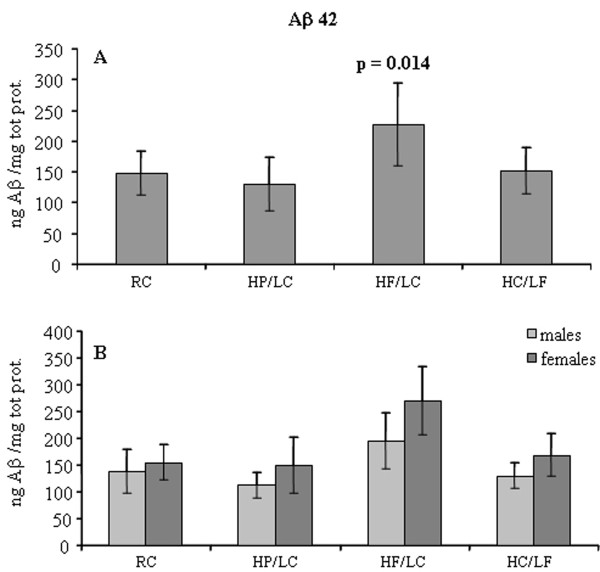
**High fat/low carbohydrate diet is associated with increased solubilizable Aβ42 levels in a murine model of aggressive AD neuropathology**. TgCRND8 mice (n = 51) were fed for 14 weeks with regular commercial chow (RC), high fat custom chow (HF), high protein custom chow (HP), or high carbohydrate custom chow (HC). [N.B., diet groups are named by either major component only (HF, HP, HC) or by an abbreviation indicating both major and minor components (HF/LC, HP/LC, HC/LF); HF or HF/LC = high fat/low carbohydrate custom chow (60 kcal% fat/30 kcal% protein/10 kcal% carbohydrate); HP or HP/LC = high protein/low carbohydrate custom chow (60 kcal% protein/30 kcal% fat/10 kcal% carbohydrate); HC or HC/LF = high carbohydrate/low fat custom chow (60 kcal% carbohydrate/30 kcal% protein/10 kcal% fat)]. At the end of this period, brains were harvested, and levels of Aβ42 were evaluated as described in Methods. Statistical significance (p = 0.014) was determined by pairwise comparison to RC. Data represent means ± S.D.

Pairwise comparison of each diet to RC revealed that solubilizable Aβ42 levels were significantly increased in mice fed with HF/LC diet (RC vs F-P 147.89 vs 227.03, p = 0.014), but no difference was found between solubilizable Aβ42 levels of mice fed with HP/LC or HC/LF diets as compared to RC (HP/LC 130.89, p = 0.862; HC/LF 152.48, p = 1.000) (Figure 1). For solubilizable Aβ40, results approached statistical significance for the HF/LC vs RC comparison (RC vs HF/LC 26.77 vs 47.94, p = 0.064), while neither HP/LC nor HC/LF diets affect solubilizable Aβ40 levels as compared to RC (HP/LC 29.83, p = 0.966; HC/LF 29.19, p = 0.971).

The observation that the commercial diet (58 kcal% carbohydrate/29 kcal% protein/13 kcal% fat) and the custom HC/LF diet (60 kcal% carbohydrate/30 kcal% protein/10 kcal% fat) yielded identical results tends to exclude the possibility that any variations were attributable to the custom formulation *per se*. The elevation in solubilizable Aβ levels in the brains of HF/LC diet-fed TgCRND8 mice was in accordance with data obtained in other laboratories that directly connected high fat/high cholesterol diets with amyloidosis and conventional measures of soluble and insoluble Aβ [6-8]. However, in our experiment, cholesterol was added to the RC, HC/LF, and HP/LC diets in order to bring those custom diets up to an identical level of cholesterol content to that present in the HF/LC diet. Therefore, elevations in solubilizable Aβ levels in HF/LC diet-fed TgCRND8 mice were not attributable cholesterol but to dietary fats *other than *cholesterol.

A two-way ANOVA for differences in plaque density showed no statistically significant effect of diet (F_(3, 23) _= 1.039, p = 0.394), sex (F_(1, 23) _= 0.277, p = 0.603), or diet*sex interaction (F_(3,23) _= 0.237, p = 0.870). This suggests that the higher levels of solubilizable Aβ that we observed in our HF/LC diet-fed mice might involve primarily non-plaque forms of Aβ (e.g., oligomeric Aβ).

**Figure 2:**
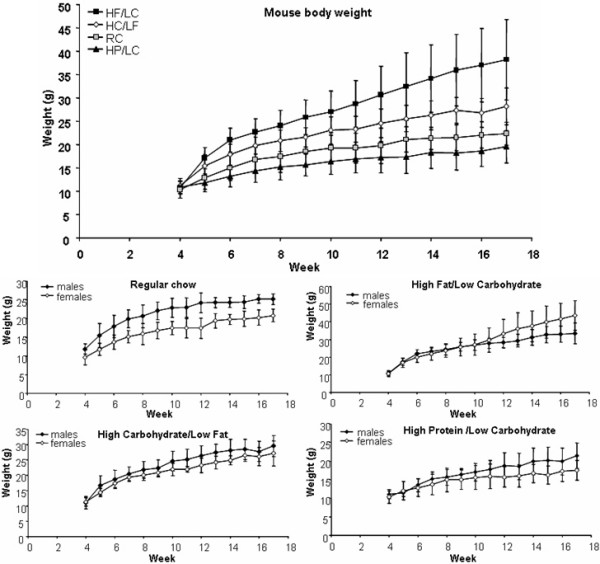
**Body weights of mice on various diets**. Body weights of TgCRND8 mice were measured weekly from week 4 until week 17 (n = 57). The figures represent the weight difference depending on different diets in the whole group (A) as well in each different diet (B). [N.B., diet groups are named by either major component only (HF, HP, HC) or by an abbreviation indicating both major and minor components (HF/LC, HP/LC, HC/LF); HF or HF/LC = high fat/low carbohydrate custom chow (60 kcal% fat/30 kcal% protein/10 kcal% carbohydrate); HP or HP/LC = high protein/low carbohydrate custom chow (60 kcal% protein/30 kcal% fat/10 kcal% carbohydrate); HC or HC/LF = high carbohydrate/low fat custom chow (60 kcal% carbohydrate/30 kcal% protein/10 kcal% fat)]. Data represent means ± S.D.

We then evaluated the effect of diets on the weight of the mice. At baseline, at the age of 4 weeks, there was no difference in body weight of the mice according to sex or diet (Table 2; Figure 2). By week 6 (i.e., only two weeks into the 4-diet protocol), we observed an effect of diet on body weight (highest to lowest: HF/LC > HC/LF = RC > HP/LC) and an effect of sex (higher weights of males than of females), but no interaction between these two factors. Pairwise comparison showed the HF/LC and HC/LF diets were significantly different from RC, while HP/LC diet was not.

**Table 2:** Two-way ANOVA of body weight by diet and gender, at weeks 4, 6, and 17, with Dunnett's pairwise comparison of RC to other diets, using Dunnett's T3 procedure for unequal variances.

	**Two-Way ANOVA**			**Pairwise comparison**	
**Week 4**	**F**	**df**	**p**		**p**

diet	0.424	3, 49	0.736	RC vs HP	0.939

gender	1.644	1, 49	0.206	RC vs HF	0.975

diet * gender	1.008	3, 49	0.397	RC vs HC	0.700

					

	Two-Way ANOVA			Pairwise comparison	

Week 6	F	df	p		p

diet	31.614	3, 19	< 0.0005	RC vs HP	0.271

gender	10.211	1, 49	0.002	RC vs HF	< 0.0005

diet * gender	0.894	3, 49	0.451	RC vs HC	0.037

					

	Two-Way ANOVA			Pairwise comparison	

Week 17	F	df	p		p

diet	52.588	3, 19	< 0.0005	RC vs HP	0.085

gender	0.003	1, 49	0.954	RC vs HF	< 0.0005

diet * gender	8.236	3, 49	< 0.0005	RC vs HC	0.001

At week 17, body weights of the mice differed by diet, but overall, there was no effect of sex (Table 2; Figure 2). A significant diet*sex interaction indicated that the weights of the male mice were higher than those of females in the RC, HP/LC, and HC/LF diets (M vs F, RC 24.7 g vs 20.8 g, HP/LC 21.4 g vs 17.8 g, HC/LF 29.6 g vs 27.1 g), while mice fed with HF/LC diet showed the opposite pattern with females being heavier than males (M vs F, HF/LC 33.5 g vs 43.7 g).

We performed a two-way ANOVA by diet and sex for repeated measures of body weight from week 6 through week 17. As shown (Table 3; Figure 2), as expected, the body weight of the mice was affected by age. There were significant age*diet and age*diet*sex interactions, while the age*sex interaction was only a trend that approached significance. ANOVA of linear trends from the repeated measures analysis showed comparable results, demonstrating that the age interactions primarily reflected linear trends. The age*diet interaction showed a greater increase in the HF/LC group than in the other groups. The age*diet*sex interaction in the HF/LC diet group showed a greater increase among females than males, while increases for males were at least as large as for females in the other diets.

**Table 3:** Repeated measures ANOVA of body weight with Huynh-Feldt correction of the degrees of freedom for violation of the sphericity assumption, and ANOVA of linear trend from the repeated measures analysis.

	**Repeated measures analysis**			**Linear trend analysis**		
	**F**	**df**	**p**	**F**	**df**	**p**

age	181.784	2.981, 146.086	< 0.0005	306.212	1, 49	< 0.0005

age * diet	14.587	8.944, 146.086	< 0.0005	23.942	3, 49	< 0.0005

age * gender	2.554	2.981, 146.086	0.058	3.495	1, 49	0.068

age * diet *gender	5.971	8.944, 146.086	< 0.0005	9.365	3, 49	< 0.0005

**Figure 3:**
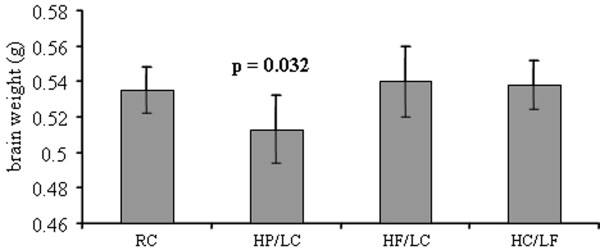
**Brain weights of mice on various diets**. Brain weights from TgCRND8 mice (n = 42) maintained on diets with various sources of calories. Statistical significance (p = 0.032) was determined by pairwise comparison to RC. [N.B., diet groups are named by either major component only (HF, HP, HC) or by an abbreviation indicating both major and minor components (HF/LC, HP/LC, HC/LF); HF or HF/LC = high fat/low carbohydrate custom chow (60 kcal% fat/30 kcal% protein/10 kcal% carbohydrate); HP or HP/LC = high protein/low carbohydrate custom chow (60 kcal% protein/30 kcal% fat/10 kcal% carbohydrate); HC or HC/LF = high carbohydrate/low fat custom chow (60 kcal% carbohydrate/30 kcal% protein/10 kcal% fat)]. Data represent means ± S.D.

We then evaluated the weights of the brains at time of death and compared them by two-way ANOVA. While diet affected the weight of the brain (F_(3,34) _= 5.413, p = 0.004), neither sex (F_(1,34) _= 0.0025, p = 0.972) nor diet*sex interaction (F_(3,34) _= 0.381, p = 0.997) affected the brain weight. A pairwise analysis showed that mice fed with HF/LC and HC/LF diets had brain weights equal to mice fed the RC diet (RC vs F-P 0.535 g vs 0.540 g, p = 0.972; vs C-P 0.538 g, p = 0.997), while mice fed the HP/LC diet had a significantly lower brain weight compared to RC (RC vs P-P 0.535 g vs 0.514 g, p = 0.032) (Figure 3). The brain/total weight ratios (brain weight as % of total weight) were [HC/LF] 1.929 ± 0.227 (SD) (n = 10); [HP/LC] 2.668 ± 0.397 (n = 13); [HF/LC] 1.413 ± 0.307 (n = 10); [RC] 2.418 ± 0.321 (n = 9). Therefore, the brain weight as % of total weight tended to be highest in the HP/LC group and lowest in the HF/LC group.

**Figure 4:**
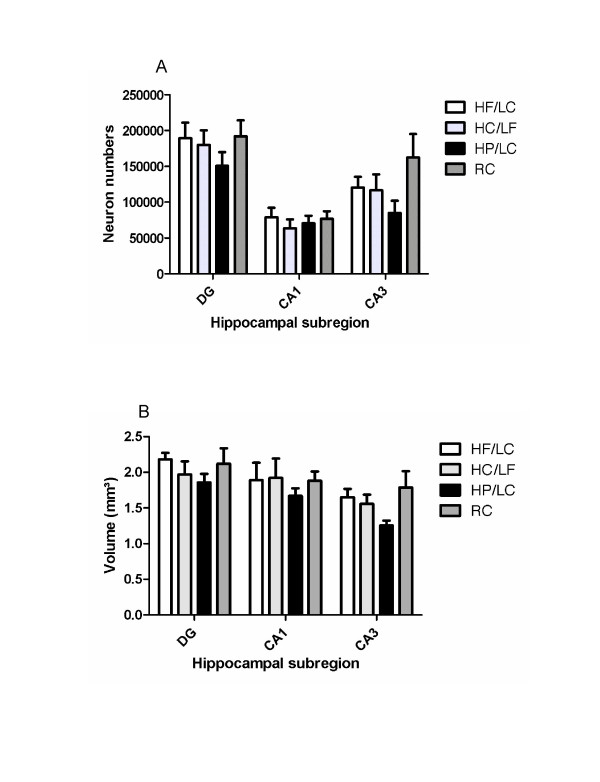
**Stereologic estimates of hippocampal subregions of TgCRND8 mice raised on various diets**. HF, high fat custom chow; HC, high carbohydrate custom chow; HP, high protein custom chow; RC, regular commercial chow. Vertical bars are means ± SEMs. A) Neuronal counts (n = 20). B) Volume estimation by Cavalieri principle (n = 22). [N.B., diet groups are named by either major component only (HF, HP, HC) or by an abbreviation indicating both major and minor components (HF/LC, HP/LC, HC/LF); HF or HF/LC = high fat/low carbohydrate custom chow (60 kcal% fat/30 kcal% protein/10 kcal% carbohydrate); HP or HP/LC = high protein/low carbohydrate custom chow (60 kcal% protein/30 kcal% fat/10 kcal% carbohydrate); HC or HC/LF = high carbohydrate/low fat custom chow (60 kcal% carbohydrate/30 kcal% protein/10 kcal% fat)].

There was a significant Pearson correlation of neuronal count and volume estimate (r = 0.420, p < 0.001) among the subset of mice studied. There were no significant effects of diet, sex, or diet*sex interaction for neuronal count or volume in any of the three hippocampal subregions: dentate gyrus, CA1, and CA3. The mean ± standard error of the mean (SEM) for each subregion is presented in Figure 4. In CA3, for neuronal count, the HP/LC mean was only 52% of the RC mean, and the HP/LC volume was only 70% of the RC volume, but the comparisons were not significant (p = 0.308 and p = 0.282, respectively). On the other hand, when HP/LC vs RC for the CA3 region were compared without adjustment for multiple comparisons, the approximate t-tests assuming unequal variances yielded p values that approached statistical significance (neuronal count: t = 2.092, df = 7.37, p = 0.073; neuronal volume: t = 2.223, df = 5.84, p = 0.069). This raises the possibility that hippocampal CA3 was involved in the loss of brain mass reflected in the diminished brain mass in HP/LC diet-fed TgCRND8 mice. However, in the absence of a cohort of HP/LC diet-fed nontransgenic mice, we cannot be certain what role, if any, was played by diet alone vs the interaction of cerebral amyloid with diet.

## Discussion and conclusion

Effects of various diets on clinical and experimental AD pathology have been reported in the literature, including (a) high carbohydrate diets, (b) restricted calorie diets, (c) the ketogenic diet, and (d) diets high or low in cholesterol and other fats. Under certain circumstances, high carbohydrate diets have been reported to exacerbate the pathology of AD in experimental animals [9]. Mice given 10% sucrose-sweetened water in order to induce glucose intolerance, hyperinsulinemia, and hypercholesterolemia, showed a 2-3-fold increase in levels of insoluble Aβ in their brains [9]. In the current study, we did not observe any association of brain weight loss, Aβ levels, or hippocampal integrity with either of two diets (one commercial, one custom) in which most calories were derived from carbohydrates [9]. A growing body of work implicates insulin resistance as a key feature of carbohydrate-related disturbances in Aβ metabolism [10], possibly explaining the apparent discrepancy between our study and that of Cao *et al*. [9], since our mice were not diabetic. A caloric restriction (CR) diet has been shown to attenuate amyloidosis in murine and monkey models [5,11].

A ketogenic diet (low in carbohydrates, high in fat) has been reported to reduce brain Aβ levels in a mouse model [12]. This is somewhat surprising since several studies in the literature show a robust association of HF/LC diet with increased solubilizable brain Aβ levels [6-8]. Here, we confirmed the generally-held association of high fat diet with increased Aβ accumulation, but, since all our diets contained identical levels of cholesterol, the solubilizable Aβ levels as measured in our HF/LC diet-fed mice must be due to fats other than cholesterol.

A typical "Western diet," containing 40% saturated fatty acids and 1% cholesterol, was reported to be associated with increased brain Aβ levels in a murine model of AD when compared with a diet enriched in docosahexaenoic acid (DHA) [13]. An omega-3 fatty acid-enriched diet was reported to reduce the amyloid burden in an AD mouse model [14]. In a clinical study of the possible associations between intake of specific types of fat and AD in a cohort of 815 65-year-old patients, intake of saturated fat and *trans*-unsaturated fat was correlated with an increased risk for clinical AD, while high intake of unsaturated, unhydrogenated fats was correlated with decreased risk [15]. Therefore, it is possible that it is specifically the saturated fat content of a diet--and not its cholesterol content -- that modulates Aβ metabolism. Some fats (e.g., DHA) are reported to be relatively protective, and it is worth noting that mice fed HF/LC diets showed no loss of brain weight or hippocampal integrity despite the higher levels of solubilizable Aβ. These data are consistent with observations that clinical dementia in humans correlates best with atrophy and not with neuropathological burden [16-21].

The most unexpected result of our study was the loss of overall brain mass associated with a HP/LC diet that, notably, did not exacerbate solubilizable Aβ levels or amyloid pathology. It is worth noting that a major confound in the field of Alzheimer's disease mouse modeling has been the conspicuous absence of neuronal loss in these transgenic mouse models. Indeed, this widely confirmed precedent led us not to include nontransgenic mice in our studies of the effects of custom diets. TgCRND8 mice, because of their aggressive amyloid pathology, are relatively insensitive to modulators of amyloidosis, and this may explain the minimal effect that we observed for dietary fat. However, the malignant amyloid pathology may well increase the sensitivity for detecting modulators of amyloid toxicity. This may explain why we observed the loss of brain mass with the HP/LC diet, but this conclusion cannot be drawn until we examine the effect of this HP/LC diet on the brains of nontransgenic mice.

The "encephalization quotient" (EQ; brain weight/body weight) is modulated by both genetic and dietary factors during brain development [22,23], but the effect of diet on EI in adult animals is less well-studied. The EQ in our study was highest among the HP/LC mice and lowest among the HF/LC group. Typically, protein deprivation is associated with low brain weight and low EQ [22]. Conversely, high fat diet is believed to have facilitated the evolution of higher EQ [23]. On the surface, then, our observations run counter to other reported effects on EQ.

Interestingly, protein-carbohydrate ratio has been demonstrated to modulate brain excitotoxicity differentially as a function of age. A high carbohydrate/low protein (HC/LP) diet has been reported to increase excitotoxicity in the hippocampus and hypothalamus of young rats, while, conversely an HC/LP diet has been associated with decreased excitotoxicity in the same brain regions in older rats. However, potentially relevant to our result, a high protein-low carbohydrate (HP/LC) diet has been reported to associate with enhanced excitotoxicity specifically in the aged brain [24]. Since Aβ is known to lower the threshold for glutamatergic excitoxicity in cultured neurons [25], it is conceivable that high protein diet could play a role in Aβ- related neurodegeneration via aging-dependent sensitization to glutamatergic excitotoxicity. Further experiments (including studies of nontransgenic mice) will be required to determine whether the trend toward lower neuronal counts and decreased volume in the hippocampal CA3 subregion might be related to Aβ toxicity and might reach statistical significance using larger cohorts. These data, if specifically associated with the presence of cerebral Aβ amyloid, suggest that a diet rich in protein might enhance neuronal vulnerability to amyloidosis.

Of course, regardless of what happens in the mouse models, the more important question is whether these data have implications for the human aging brain and/or the human AD brain. Given the association of high protein diet with aging-related neurotoxicity [24], one wonders whether particular diets, if ingested at particular ages, might increase susceptibility to incidence or progression of AD. This can only be established by prospective randomized double blind clinical trials of various diets. This would be a challenging undertaking but potentially worthwhile if there is a serious possibility for modifying the course of AD by via manipulating dietary composition.

## Competing interests

S.G. holds an investigator initiated grant from Forest Research Institute, and he also holds consultancy positions with Diagenic and with Amicus Therapeutics. S.G. serves on the Safety Monitoring Board for the Wyeth/Elan Aβ active vaccination program.

## Authors' contributions

SP, CT, and HB performed the experiments and statistical analysis, and JS assisted with the statistical analysis. LH, RM, GP, and SG designed the custom diets. PF, DW, and PSH created and characterized the TgCRND8 transgenic mice. JDB provided reagents and advice for the Aβ analyses. DLD and PRH supervised the morphometric analyses. ME and SG provided the overall design, supervised the collection and analysis of data, secured funding for the project, and supervised the preparation of the manuscript.
